# Effects of Different Types of Fibers on the Physical and Mechanical Properties of MICP-Treated Calcareous Sand

**DOI:** 10.3390/ma14020268

**Published:** 2021-01-07

**Authors:** Jitong Zhao, Huawei Tong, Yi Shan, Jie Yuan, Qiuwang Peng, Junling Liang

**Affiliations:** 1 School of Civil Engineering, Guangzhou University, Guangzhou 510006, China; 2111816008@e.gzhu.edu.cn (J.Z.); tonghw@gzhu.edu.cn (H.T.); yshan@gzhu.edu.cn (Y.S.); 2111916143@e.gzhu.edu.cn (J.L.); 2 Foshan Railway Investment Construction Group Co., Ltd., Foshan 528000, China; pengqiuwang@fmetro.net

**Keywords:** microbial-induced calcite precipitation (MICP), water absorption, unconfined compressive strength (UCS), glass, polyester, and hemp fibers, bonding effect

## Abstract

Microbial-induced calcite precipitation (MICP) has been a promising method to improve geotechnical engineering properties through the precipitation of calcium carbonate (CaCO_3_) on the contact and surface of soil particles in recent years. In the present experiment, water absorption and unconfined compressive strength (UCS) tests were carried out to investigate the effects of three different fiber types (glass fiber, polyester fiber, and hemp fiber) on the physical and mechanical properties of MICP-treated calcareous sand. The fibers used were at 0%, 0.10%, 0.15%, 0.20%, 0.25%, 0.30%, 0.35%, and 0.40% relative to the weight of the sand. The results showed that the failure strain and ductility of the samples could be improved by adding fibers. Compared to biocemented sand (BS), the water absorption of these three fiber-reinforced biocemented sands were, respectively, decreased by 11.60%, 21.18%, and 7.29%. UCS was, respectively, increased by 24.20%, 60.76%, and 6.40%. Polyester fiber produced the best effect, followed by glass fiber and hemp fiber. The optimum contents of glass fiber and polyester fiber were 0.20% and 0.25%, respectively. The optimum content of hemp fiber was within the range of 0.20–0.25%. Light-emitting diode (LED) microscope and scanning electron microscope (SEM) images lead to the conclusion that only a little calcite precipitation had occurred around the hemp fiber, leading to a poor bonding effect compared to the glass and polyester fibers. It was therefore suggested that polyester fiber should be used to improve the properties of biocemented sand.

## 1. Introduction

Calcareous sand is the main foundation soil in the South China Sea. It is a type of special rock and soil medium with irregular shapes, many internal pores, low load-bearing capacity, and ready susceptibility to particle breakage under pressure [1]. This soil foundation is easily prone to destruction due to insufficient tolerance capacity under the action of periodic load [2]. Therefore, it requires foundation treatment to improve the physical and mechanical properties of the calcareous sand area [3].

A new soil reinforcement technology has been found to improve the physical and mechanical properties of calcareous sand, known as microbial-induced calcite precipitation (MICP) [4,5]. It was first proposed by Australian geological engineer Whiffin [6]. The MICP treatment method has gained interest due to its relatively environmentally friendly characteristics, low energy consumption, and sustainable advantages [7]. Many recent studies have shown that the MICP treatment technique could effectively improve strength and stiffness [8,9,10,11,12]; decrease permeability [13,14,15,16]; increase resistance to liquefaction [17,18,19]; and enhance concrete self-healing [20,21,22]. All the above studies indicated that as a new technology for foundation reinforcement, MICP could effectively improve stiffness, strength, and permeability. It was also found to offer a potential value when applied to improve liquefaction and concrete self-healing.

A few studies among those related to improving sand by MICP treatment investigated reinforced sand ductility by adding fibers [23,24,25,26]. Among these, Choi [24] mixed Ottawa 20–30 sand and polyvinyl acetate (PVA) fibers in five different fiber proportions, and found that the splitting tensile strength and splitting secant elastic modulus increased when the ratio of either calcium carbonate or fiber content was augmented. Xiao [25] obtained unconfined compressive strength (UCS), splitting tensile strength (STS), and peak failure state strain increases by increasing fiber content relative to a given calcite content. This was interpreted to be due to the interlocking, reinforcing, and bonding effects observed in SEM images.

However, the above studies were all based on the effect of adding a single type of fiber into the biocement material. The present experiment aimed to fully understand the effects of different fiber types on the physical and mechanical properties of MICP-treated calcareous sand. According to different sources, fibers can be roughly divided into natural, synthetic, and inorganic fibers in nature. Inorganic fiber refers to a chemical fiber made by a chemical reaction with minerals as a raw material. Its difference from synthetic fiber is based on the different sources of raw materials. Common inorganic fibers mainly include glass fiber, ceramic fiber, metal fiber, carbon fiber, etc. Synthetic fiber, also known as chemical fiber, refers to the polymer produced by a chemical reaction to form a polymer. There are various types of synthetic fiber, which often show different physical and chemical properties due to the different raw materials of the polymer. Natural fibers refer to the fibers on plants and animals that are naturally generated in nature. According to the sources of natural fibers, they can be divided into two categories: plant fibers and animal fibers. As a kind of fiber that widely exists in nature, they are widely used in various industries because of their low cost, easy access, and high yield. In this paper, we selected three fiber types commonly used in previous fiber-reinforced soil studies: glass fiber [27,28,29], polyester fiber [30,31], and hemp fiber [32,33]. For this purpose, a series of water absorption and unconfined compressive strength (UCS) tests were carried out to investigate the effects of these three different types of fibers. This study aims at providing a theoretical basis for the selection of fibers for field MICP-treated sand.

## 2. Materials and Methods

### 2.1. Materials

#### 2.1.1. Bacterial Culture and Cementation Solution

A sterilized liquid medium consisting of 20 g/L yeast extract, 10 g/L (NH_4_)_2_SO_4_, and 1.6 g/L NaOH (Xilong Scientific Co., Ltd., Shantou, China) with a pH of 9.0 was used for the bacterial (*Sporosarcina pasteurii*, GDMCC China) fermentation. The bacteria were cultured in a conical flask at 200 rpm and 28 °C for 36 h. The bacteria had an optical density of 1.0 to 1.8. The urease activity of the bacteria was measured by way of conductivity-measuring medium, and was approximately 4.75 mM urea/min. The cementation solution was composed of 0.5 mol/L CaCl_2_ and 0.5 mol/L urea [34].

#### 2.1.2. Characteristics of Calcareous Sand

**Figure 1:**
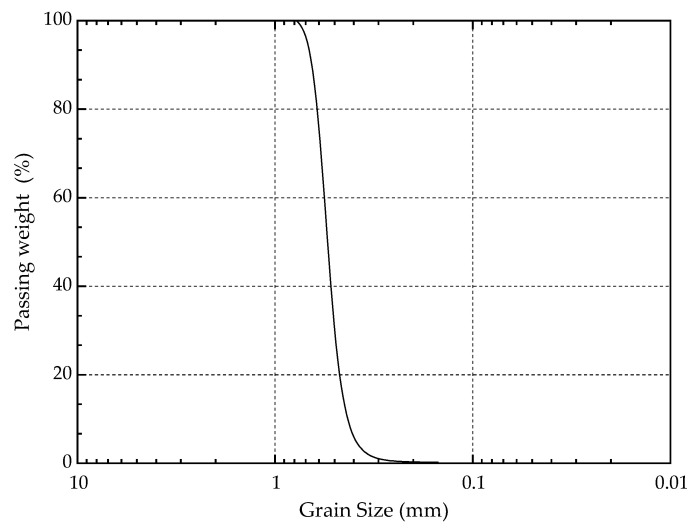
Grain size distribution of the calcareous sand.

Calcareous sand from Yongxing Islands located in the South China Sea was chosen for the tests. Its physical and mechanical parameters are shown in Table 1 below. Before the test, the sand was placed in an oven for drying at 40 °C for 24 h. The grading curve of calcareous sand is shown in Figure 1 below. The grain size of the sand ranged from 0.2 to 1 mm, which was classed as fine sand with uniform sand particles and poor grading.

#### 2.1.3. Fiber Types

**Figure 2:**
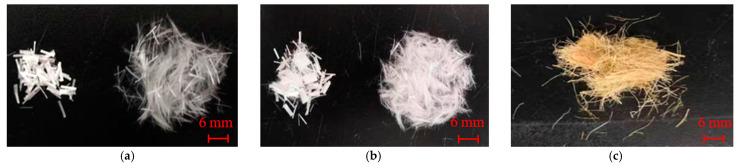
Morphology of the fibers: (**a**) Glass fiber; (**b**) Polyester fiber; (**c**) Hemp fiber.

In the experiments, glass fiber, polyester fiber, and hemp fiber were selected. Silicon dioxide (SiO_2_) dominates the chemical composition of glass fiber, giving the glass fiber better resistance to wear and corrosion [35]. The polyester fibers had a special trilobal cross-section which offers better surface roughness than the conventional circular cross-section [30]. Hemp fiber belongs to the natural plant fiber category, which is more widely distributed and easier to obtain than animal fibers. The physical and mechanical properties of these three different fiber types used in the experiments are shown in Table 2 below; the morphology of these three fibers is shown in Figure 2 below.

### 2.2. Specimen Preparation

#### 2.2.1. The Clean and Fiber-Reinforced Sand

Cylindrical specimens with a diameter of 39.1 mm and a height of 80 mm were used for the water absorption and unconfined compressive strength tests. The relative density of the sample was controlled at 50% (130.67 g of calcareous sand) [36]. The three different fibers at 0.1%, 0.15%, 0.2%, 0.25%, 0.3%, 0.35%, and 0.4% (by weight of sand, respectively) were added. Furthermore, a control group without any fiber was set up. Eight percent of distilled water (by weight) was added to the sand and easily mixed uniformly with the fiber within the sand column. After uniform stirring, the specimens were divided into three layers into the mold. Each layer was slightly compacted with a compaction tool.

#### 2.2.2. Grouting Procedure

**Figure 3:**
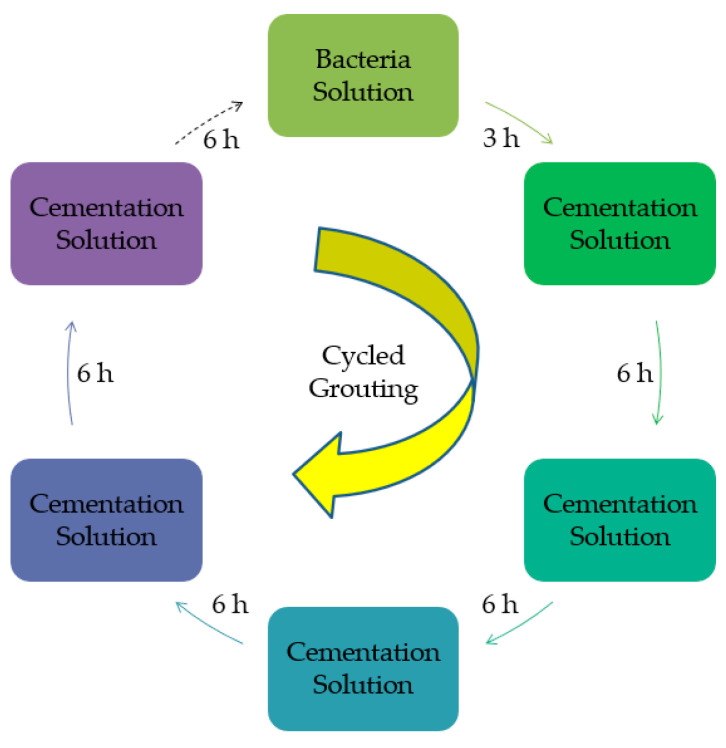
Grouting procedure.

Based on the study of Xiao [19] and our instrument requirements, a peristaltic pump was used in the experiments to reinforce samples with cycled grouting. The grouting reinforcement procedure is shown in Figure 3 below.

**Figure 4:**
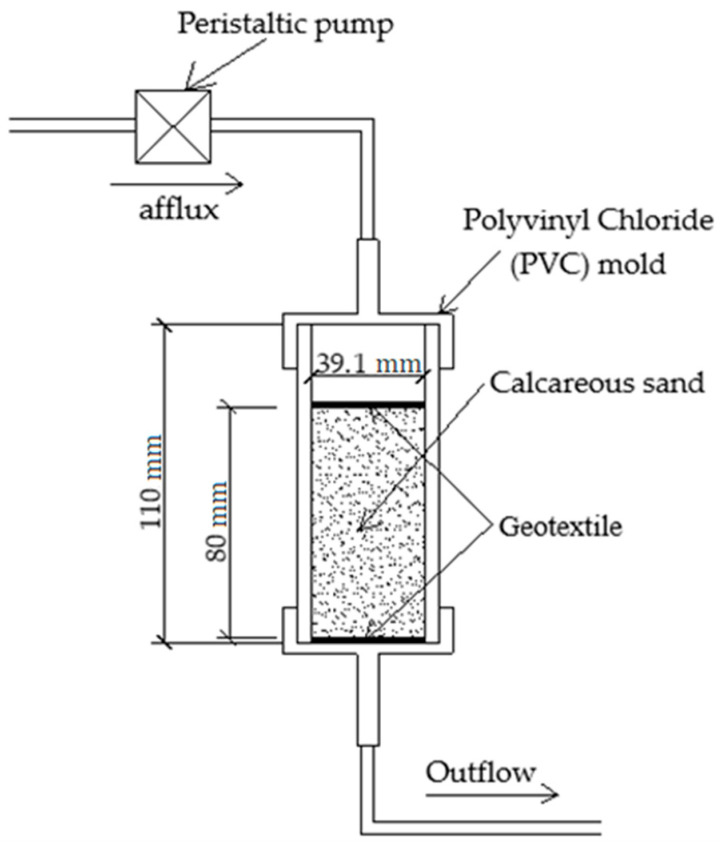
Schematic diagram of the grouting procedure.

**Figure 5:**
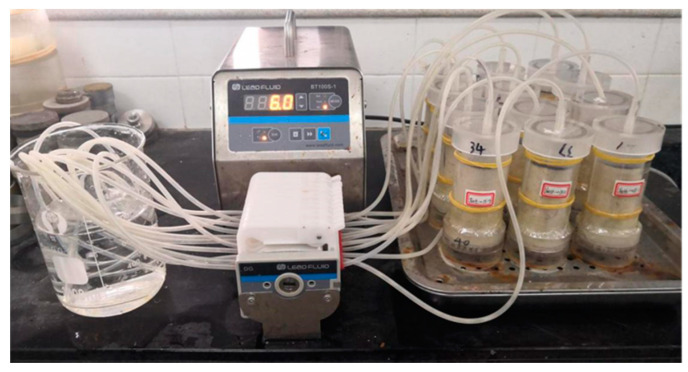
Grouting procedure of the peristaltic pump.

The grouting rate was set at 1.5 mL/min. A total of 4 rounds of grouting were carried out, and each injection volume was one specimen pore volume (i.e., 50 mL). The specimens were prepared at a room temperature of approximately 25 °C [37]. The schematic diagram of the grouting procedure is shown in Figure 4 below. A photograph of the grouting is shown in Figure 5 below.

### 2.3. Testing Method

#### 2.3.1. Water Absorption Test

The water absorption represented the water absorption capacity under standard atmospheric pressure of the specimen after grouting. It also reflected the quantity of surface pores of the specimen. Therefore, the water absorption capacity of biocemented sand was studied using the water absorption test. Based on Manzur’s research [38], the water absorption capacity of the biocemented sand was tested. The test steps were as follows: the MICP-treated specimens were dried in an oven (Yicheng Instrument Manufacturing Co. Ltd., Shaoxing, China) at 108 °C, and the dry mass m_1_ was recorded. Then, the specimen was dipped to a depth of 5–10 mm in distilled water for 24 h and removed from the sand column. Excess water was blotted off, and the new mass m_2_ was recorded. Lastly, the water absorption of the specimen ω was calculated according to Equation (1):

(1) $$\omega =\frac{m_{2}-m_{1}}{m_{1}}\times 100\%$$

#### 2.3.2. Unconfined Compressive Strength Test

A series of unconfined compressive tests was performed to measure the unconfined compressive strength (UCS) of the specimens after grouting. The specimens used in the UCS test were dried in an oven at 108 °C. The instrument used for the test was a YAW-S300 automatic liquid crystal pressure testing machine. A loading rate of 1 mm/min was adopted. Table 3 below lists the detailed test results of 22 specimens with different fiber types and contents.

## 3. Results

### 3.1. Water Absorption Test Results

**Figure 6:**
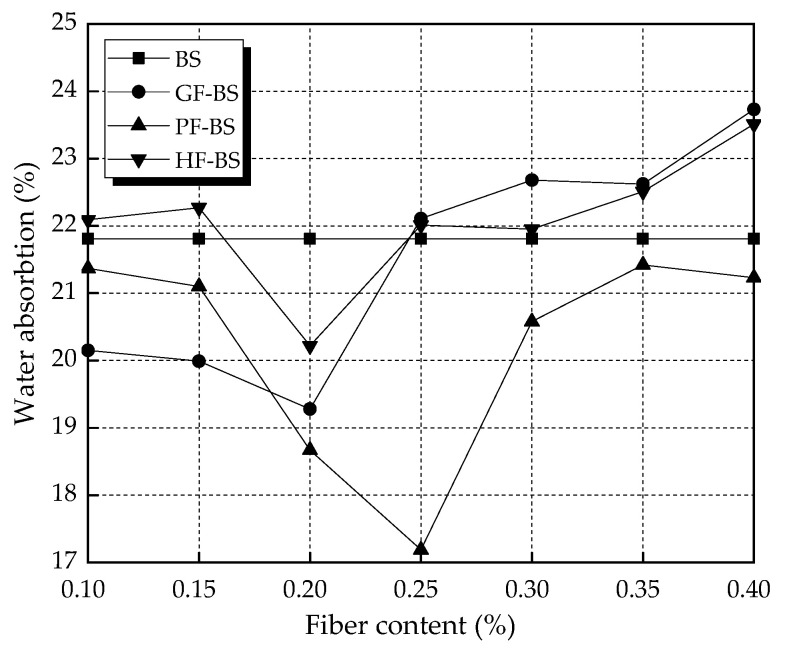
Water absorption of the specimens.

The effects of water absorption by the specimens are given in Figure 6 below. For all fiber types, water absorption decreased with increased fiber content. This trend continued up to a certain fiber content, beyond which water absorption increased. The minimum values of water absorption by the glass fiber biocemented sand (GF-BS), polyester fiber biocemented sand (PF-BS), and hemp fiber biocemented sand (HF-BS) were obtained at 0.20%, 0.25%, and 0.20% fiber content. This showed a reduction of 11.60%, 21.18%, and 7.29%, respectively, in water absorption over the BS control. The water absorption of the GF-BS was higher than the BS only under high fiber contents, while that of HF-BS essentially remained above the BS except for the 0.20% fiber content. When comparing the water absorption of three different fiber-reinforced biocemented sand samples, it was found that the water absorption of PF-BS was the lowest, followed by GF-BS and HF-BS.

### 3.2. Stress-Strain Curve of UCS Test Results

**Figure 7:**
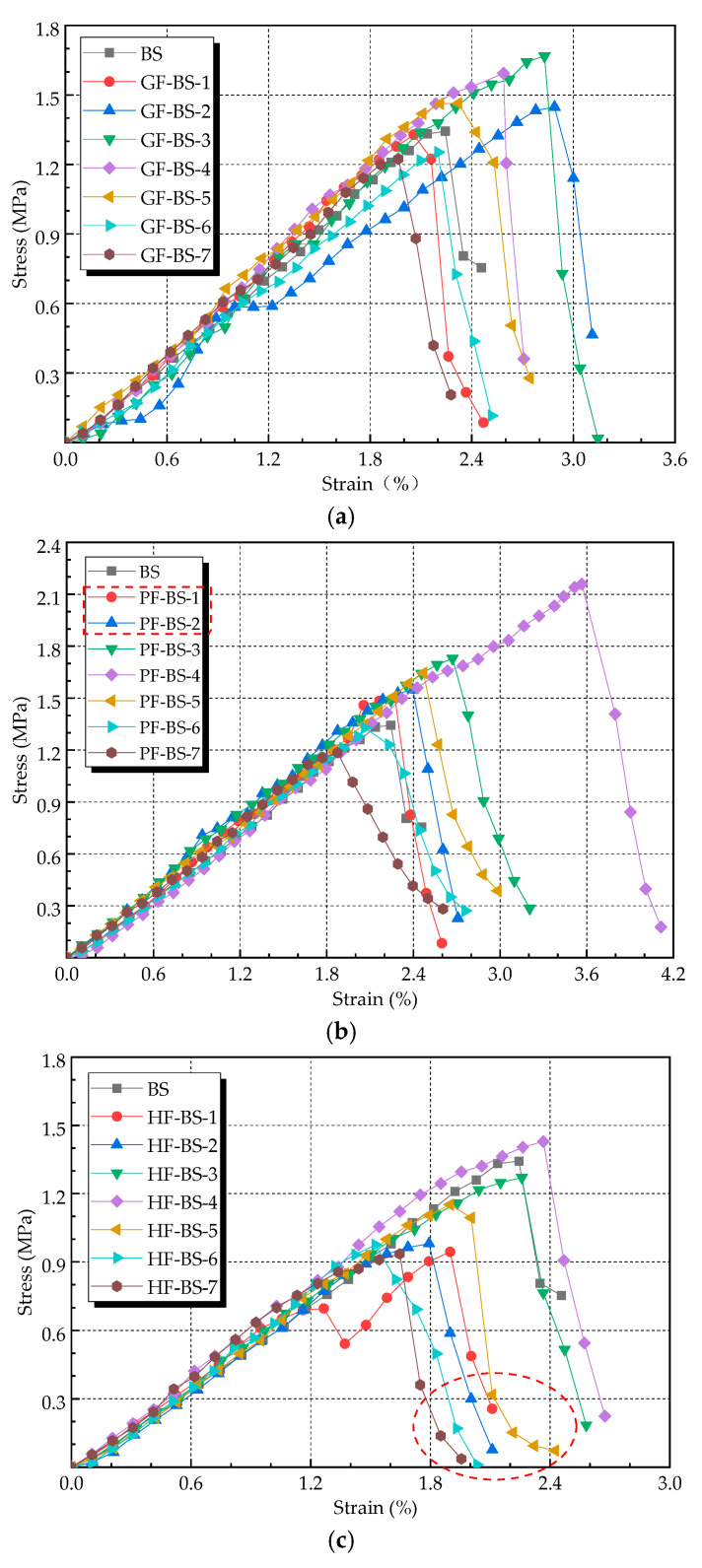
Stress-strain curves of reinforced sand under different fiber types and contents: (**a**) stress-strain curves of the GF-BS; (**b**) stress-strain curves of the PF-BS; (**c**) stress-strain curves of the HF-BS.

The results of the stress-strain curve of three different fiber-reinforced biocemented sand samples are shown in Figure 7. With the vertical load, the stress increased with the rise in strain in the initial stage until the stress reached its peak. This was similar to the elastic deformation stage. The biocemented sands under different fiber types and contents showed similar behavior at the initial stage until the stress reached its peak. As the stress of the biocemented sands reached its peak value, the samples began to crack and become damaged. For the GF-BS, the stress and strain trend of the sample after failure was roughly consistent with that of the BS, as shown in Figure 7a below. It indicated that glass fiber was ineffective in improving the ductility of the sample. For the PF-BS, as shown in Figure 7b below, at the low fiber content of 0.1% and 0.2%, poor improvement in ductility of the samples was achieved. However, under other fiber contents, the stress-strain trend of samples after failure was slower than that of the BS. This indicated that when the polyester fiber content was 0.2% to 0.4%, sample ductility could be increased. For the HF-BS, the ductility improvement of the sample was relatively poor: the decreasing trend of stress and strain became slow only when the stress value of the sample decreased to a low level, as shown in Figure 7c below. This indicated that the hemp fiber cannot play an immediate role when the sample is destroyed. In conclusion, polyester fiber had the best effect on the improved ductility of the biocemented sand.

### 3.3. Unconfined Compressive Strength Test Results

**Figure 8:**
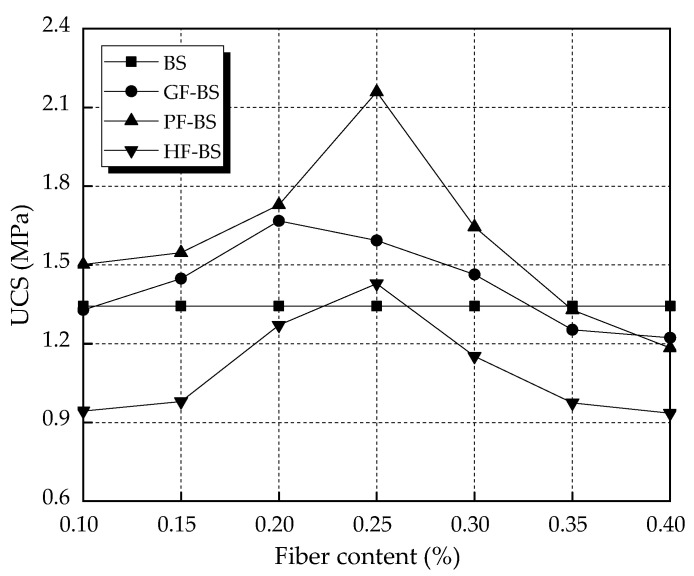
Unconfined compressive strength of biocemented sands.

The results of unconfined compressive strength (UCS) tests of biocemented sands are given in Figure 8 below. For the fiber-reinforced biocemented sand, the UCS increased with the increase in fiber content. When the fiber content increased to a certain value, UCS decreased as the fiber content increased. The maximum UCS of the GF-BS, PF-BS, and HF-BS was obtained at 0.20%, 0.25%, and 0.25% fiber content. The maximum strengths were 1.668 MPa, 2.159 MPa, and 1.429 MPa, respectively. This showed an increase of 24.20%, 60.76%, and 6.40% in strength, respectively, compared to the BS. The UCS of the GF-BS and HF-BS were lower than BS only under high contents. Comparing the UCS of three different fiber-reinforced biocemented sands led to the conclusion that the UCS of PF-BS was the highest. The UCS of GF-BS was the second highest, while the UCS of HF-BS was the lowest. On the basis of results from a comprehensive water absorption test, it was concluded that the optimum content of glass fiber and polyester fiber were 0.20% and 0.25%, respectively. The optimum content of hemp fiber was shown to be within the range of 0.20–0.25%.

## 4. Discussion

### 4.1. UCS Versus Water Absorption

**Figure 9:**
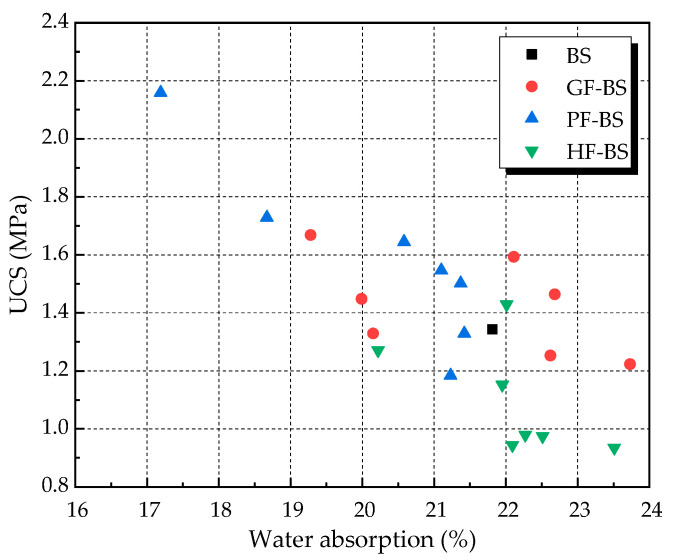
UCS and water absorption.

Figure 9 below shows a UCS and water absorption diagram and includes the results of all biocemented sand tests. On the whole, it can be seen that with increased water absorption, the UCS of the sample decreased. This indicated that the pores on the surface of the sample were detrimentally impacting on the strength of the sample. The surface pores of the sample were determined by the location of calcium carbonate precipitation, which in turn depended on the path the solution flowed through during grouting. If the solutions could be made to flow into the sample more uniformly during grouting, the number of surface pores of the sample would be relatively reduced. This was beneficial to improve the strength of the sample.

### 4.2. The Modulus of Elasticity

With vertical loading, stress increased at the initial stage alongside strain until the stress achieved its peak. This was similar to the elastic deformation stage. The biocemented sands under different fiber types and contents displayed the same behavior at the initial stage. Therefore, the stress-strain curve before peak stress was considered as a straight line. The modulus of elasticity of the sample was the slope of the line. The modulus of elasticity of all samples is shown in Table 3 below.

**Figure 10:**
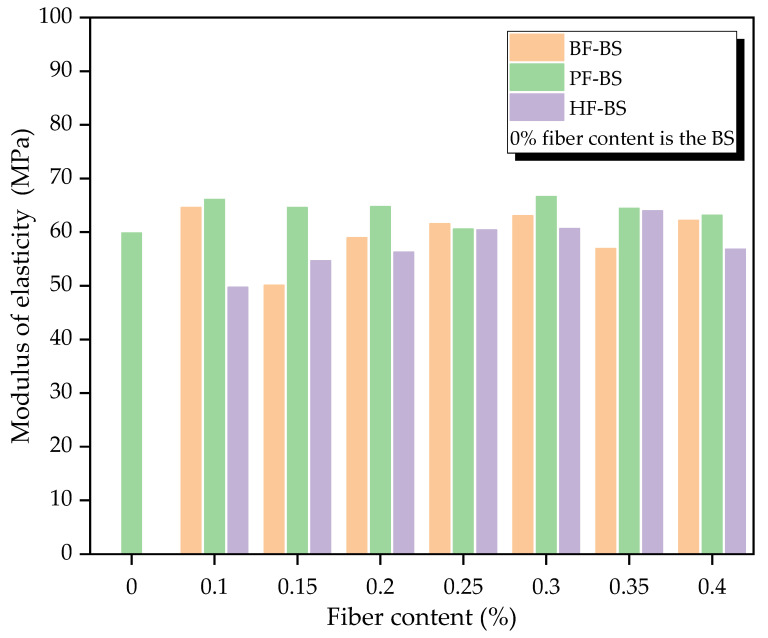
Modulus of elasticity of biocemented sand.

Figure 10 below shows the histogram obtained from the data in Table 3. As can be seen from Figure 10, the modulus of elasticity of PF-BS varies little under different fiber contents. GF-BS and HF-BS showed a general tendency to increase first and then decrease with the augmentation of fiber content. Comparing the three types of fiber-reinforced biocemented sands with different fiber contents, the modulus of elasticity of the PF-BS was higher than that of the other two types of fiber-reinforced biocemented sands. Both elastic moduli of PF-BS were higher than that of the BS. The modulus of elasticity of GF-BS was generally greater than that of HF-BS. The magnitude of the modulus of elasticity of all three types of fiber-reinforced biocemented sands was the same as that of unconfined compressive strength. This indicated that the stress of PF-BS was the highest under the same strain condition. In other words, the PF-BS achieved the greatest stiffness. The performance of the elastic modulus of PF-BS may have been due to its high calcium carbonate precipitation and the high tensile strength and modulus of elasticity of the polyester fiber. Therefore, the polyester fiber had the greatest effect on MICP-treated sands. The modulus of elasticity of the PF-BS was the highest, followed by the GF-BS and HF-BS.

### 4.3. Analysis of the Failure Pattern of Biocemented Sand

**Figure 11:**
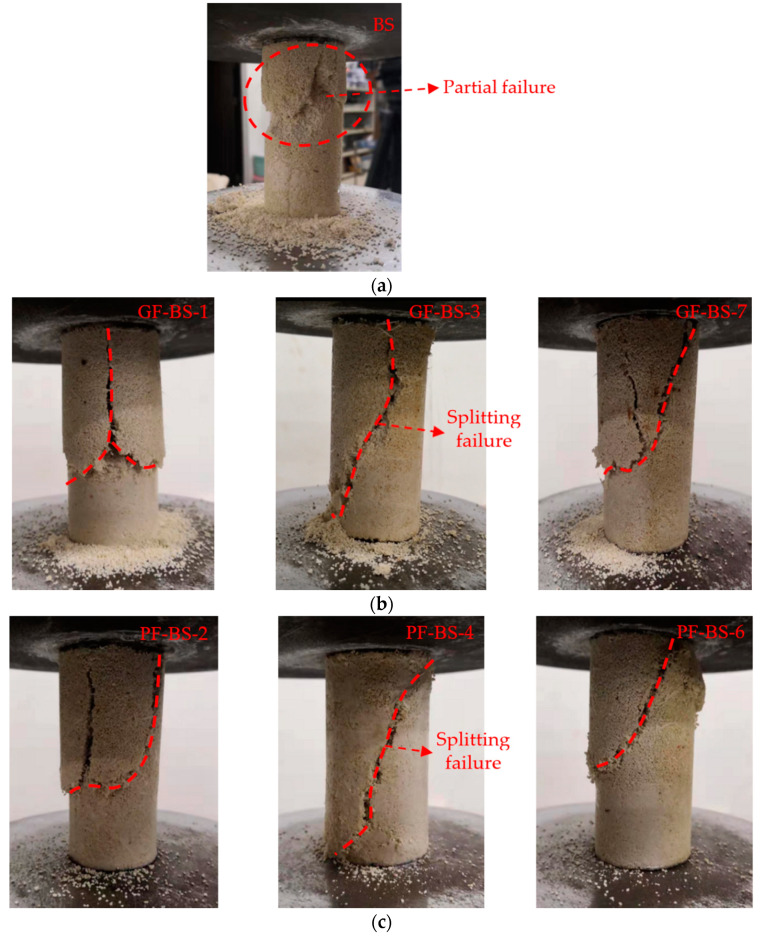

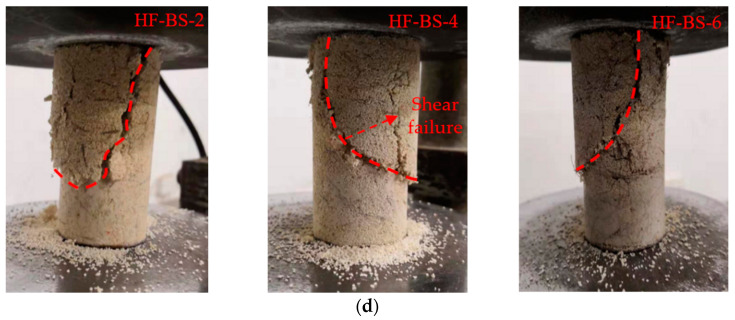
Failure pattern of biocemented sand under different fiber types and contents, (**a**) Failure pattern of the BS; (**b**) Failure pattern of biocemented sand under different glass fiber contents; (**c**) Failure pattern of biocemented sand under different polyester fiber contents; (**d**) Failure pattern of biocemented sand under different hemp fiber contents.

The failure pattern of biocemented sand is shown in Figure 11 below. As shown in Figure 11 (BS), with the action of vertical pressure, sand particles at a certain position on the edge of the sample would begin to desquamate and gradually extend to the interior of the sample. The BS was eventually damaged by the partial failure. The samples under the different fiber types and contents were mainly damaged by shear failure (Figure 11 (HF-BS-4)) and splitting failure from top to bottom (Figure 11 (GF-BS-3), (PF-BS-4)). The reason was that when the weak connection in the sample approached breaking point, the fiber connection began to act across the fracture. The fiber connection across the fracture was found to have the ability to improve the partial strength of the sample. Moreover, this fiber connection provided the fiber-reinforced biocemented sand with a residual bearing capacity after cracking, thus the ductility of the sample was improved.

The samples with glass fiber at a 0.20% content (Figure 11 (GF-BS-3)) and polyester fiber at a 0.25% content (Figure 11 (PF-BS-4)) were damaged by splitting failure. However, the sample mixed with hemp fiber at a 0.25% content (Figure 11 (HF-BS-4)) was damaged by shear failure at the top of the sample. This meant that the failure forms of the samples mixed with glass or polyester fiber were damaged by splitting failure at the maximum unconfined compressive strength, while the hemp fiber sample was damaged by shear failure. Moreover, the glass fiber and polyester fiber samples also incurred shear failure at low or high contents. This indicated that the glass fiber and polyester fiber samples provided greater improvement of the properties of the biocemented sand samples. Under high and low fiber contents, the failure type of the samples was changed from splitting failure to shear failure.

### 4.4. Microscopic Analysis

**Figure 12:**
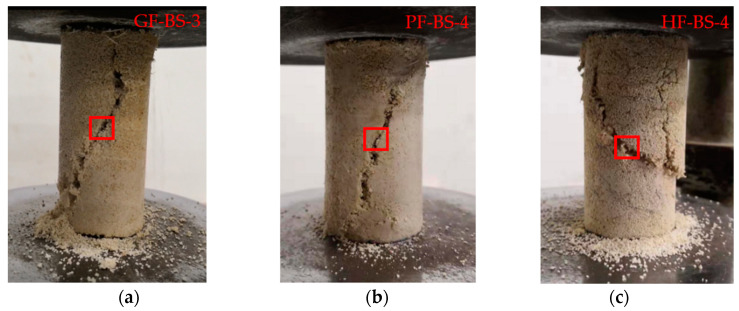
The specimens’ position was used for microscopic analysis: (**a**) GF-BS-3; (**b**) PF-BS-4; (**c**) HF-BS-4.

The specimens used for microscopic analysis were chosen from the samples with the highest UCS. As shown in Figure 12 below, specimens were obtained from the middle of the specimen and a position on the plane of fracture. Since the grouting was poured down from the top of the sample, this method was able to control the effects of the duration of the solution being poured through and the effect of the magnitude of UCS.

#### 4.4.1. Digital LED Microscope Scanning

**Figure 13:**
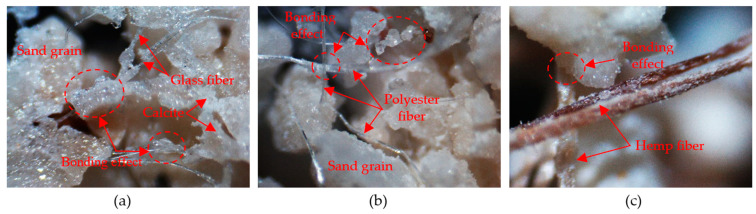
Digital LED microscope images of sand after microbial-induced calcite precipitation (MICP) reinforcement (50× magnification) at (**a**) 0.2% glass fiber, (**b**) 0.25% polyester, and (**c**) 0.25% hemp fiber.

Figure 13 below shows digital LED microscope images of sand specimens with different fibers. Figure 13a,b shows that a large amount of calcite had precipitated onto the grain surfaces, grain contacts, fiber surface, and grain-fiber contacts. Specifically, calcite on the fiber surface and grain-fiber contacts led to greater connections in the biocemented sands between fiber and sand grain, termed the “bonding effect”, as shown in Figure 13. The bonding effect affected the increase in strength. As shown in Figure 13c, the bonding effect of hemp fiber sand was less than that of glass fiber (Figure 13a) and polyester fiber (Figure 13b). This is one of the reasons why hemp fiber is ineffective in MICP-treated calcareous sand.

#### 4.4.2. SEM Scanning

**Figure 14:**
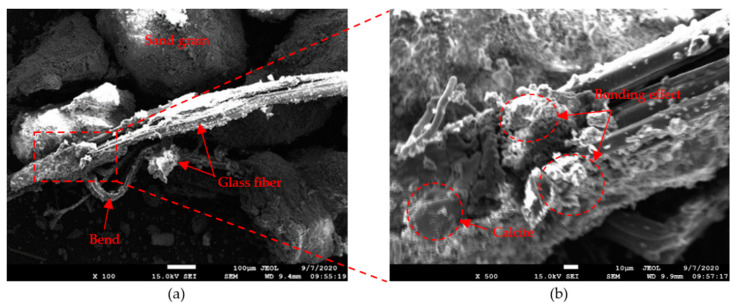

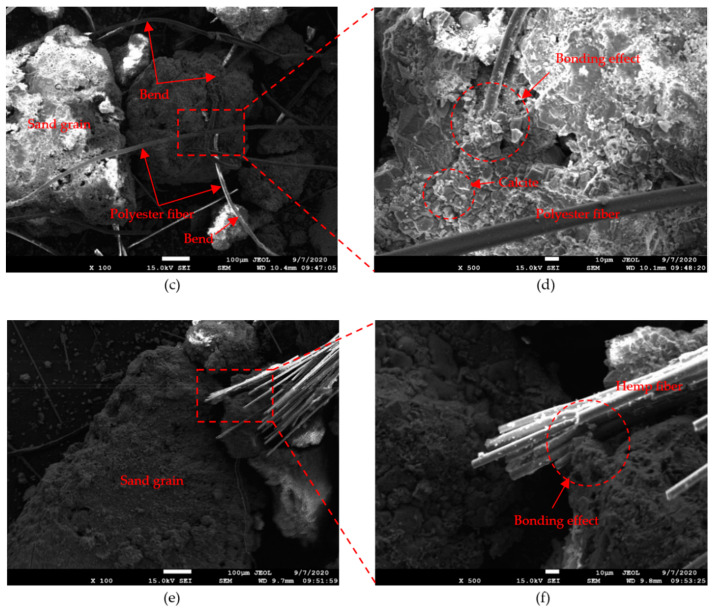
SEM images of sand after MICP reinforcement of 0.2% glass fiber at (**a**) 100× and (**b**) 500× magnification; 0.25% polyester fiber at (**c**) 100× and (**d**) 500× magnification, and 0.25% hemp fiber at (**e**) 100× and (**f**) 500× magnification.

Figure 14 below shows the SEM images of the biocemented sand mixed with different fibers. As shown in Figure 14, the calcite on the fiber and sand contacts has formed a bonding effect between fiber and sand grains. This is the key factor for the fiber to play a role in biocemented sand. Because of this bonding effect, the ductility and strength of fiber-reinforced biocemented sands were improved. As shown in Figure 14e,f, little calcite had precipitated around the hemp fiber, leading to a poor bonding effect. The unconfined compressive strength of sand mixed with hemp fiber was relatively low, and some contents were even lower than the strength of the BS. Calcium carbonate precipitation of the HF-BS in Table 3 was also less than that of GF-BS and PF-BS. Therefore, it was speculated that this may have been due to hemp fiber being smooth compared to glass and polyester fibers. Hemp fiber had a detrimental effect on the retention of bacterial and cementation solution during grouting. This resulted in reduced calcite precipitation. Figure 14 also shows that after the specimens were damaged, the hemp fibers did not bend noticeably (Figure 14f), while the glass fiber (Figure 14b) and polyester fiber (Figure 14d) did. As a result, the strength of hemp fiber was not fully utilized. Therefore, when the specimens were damaged, the bonding effect between fiber and sand grains was weakened, resulting in reduced strength of the HF-BS.

### 4.5. Discussion on the Optimum Fiber Content

In some previous studies of fiber-reinforced soil, the results of optimum fiber content were also obtained [39,40,41]. Based on the test results of water absorption and unconfined compressive strength, the optimum content of glass fiber and polyester fiber obtained were 0.20% and 0.25%, respectively. The optimum content of hemp fiber was within the range of 0.20–0.25%. The reason was that when preparing the sample, the relative density was controlled at 50% and the height of the sample was controlled at 80 mm, so the pore volume of the sample was consistent. The fibers added would occupy the original pores of the sample and would therefore also occupy the living space of the bacteria. Adding excessive fiber would have compressed the spatial environment of bacterial growth. It could have led to restrained microbial growth and had a negative effect on the precipitation of calcium carbonate. As a result, the strength of the sample would have been reduced. Other similar studies have previously been carried out. Xie [42] added polypropylene fiber to silica sand and microbially reinforced the sand. By unconfined compressive strength and calcium carbonate content tests, Xie concluded that increased fiber content augmented the pore volume occupied by fiber. The growth environment space originally belonging to microorganisms was reduced, which led to further restraining of microbial growth. The increase in fiber content also produced a negative effect on the deposition of calcium carbonate. Qiu [43] added carbon fiber to silica and calcareous sand and microbially reinforced the sand. Qiu carried out a series of calcium carbonate content, unconfined compressive strength, and permeability coefficient tests. He concluded that the void in the sand samples was limited, and that the volume of space occupied by fiber increased significantly with higher fiber content. The original growth space of the microorganisms was forced to compress, resulting in a negative effect on the growth of microorganisms. It could also be seen from the calcium carbonate precipitation ∆m in Table 3 in this paper that when fiber content increased to a certain value, ∆m decreased. This suggested that adding excessive fiber would compress the growth environment space of bacteria and restrain calcium carbonate precipitation.

## 5. Conclusions

Series of water absorption and UCS tests and SEM scanning on three different fiber-reinforced biocemented sands were conducted to investigate the effectiveness of three different types of fibers on MICP-treated calcareous sands. The following conclusions can be drawn from the study:

1. For fiber-reinforced biocemented sand, water absorption decreased with increased fiber content. When fiber content rose to a threshold, the water absorption increased as fiber content increased. The minimum water absorption obtained for the GF-BS, PF-BS, and HF-BS was at 0.20%, 0.25%, and 0.20% of fiber content, respectively. This showed a reduction of 11.60%, 21.18%, and 7.29%, respectively, in water absorption over the BS. The unconfined compressive strength displayed the opposite trend. The maximum UCS of the GF-BS, PF-BS, and HF-BS obtained was at 0.20%, 0.25%, and 0.25% of fiber content, respectively. The maximum strengths were 1.668 MPa, 2.159 MPa, and 1.429 MPa. This showed an increase of 24.20%, 60.76%, and 6.40%, respectively, in strength over the BS. On the whole, with the increase in water absorption, the UCS of the sample decreased. This indicated that the pores on the surface of the sample had a negative effect on the strength of the sample.
2. For the PF-BS, when the polyester fiber content was 0.2% to 0.4%, the ductility of the sample could be increased. For the HF-BS, only when the stress value of the sample decreased to a low level, did the decreasing trend of the stress and strain become slow. This indicated that the hemp fiber was unable to play an immediate role when the sample was destroyed. For the modulus of elasticity of these three fibers, the PF-BS was the highest, followed by the GF-BS and HF-BS. This indicated that the stress of PF-BS was the highest under the same strain conditions. In other words, the PF-BS achieved the strongest stiffness.
3. The failure patterns of different fiber-reinforced biocemented sands indicated that the glass fiber and polyester fiber were more effective in improving the properties of the biocemented sand. The failure form of the samples mixed with these two types of fibers was damaged by splitting failure at the maximum unconfined compressive strength, while the hemp fiber sample was damaged by shear failure. The BS was damaged by partial failure. Therefore, the fiber-reinforced biocemented sands displayed better integrity after damage.
4. The digital LED microscope and SEM scanning images demonstrated that the calcite on the fiber and sand contacts formed a bonding effect between fiber and sand grains, which was identified as the key factor for fiber to play a role in biocemented sands. In contrast, in the case of hemp fiber, little calcite precipitation was noted around it, leading to a poor bonding effect. It was speculated that hemp fiber was smooth compared with glass and polyester fiber, which had a detrimental effect on the retention of bacterial and cementation solution during grouting, so the strength of the HF-BS was reduced.

Overall, the optimum contents of glass fiber and polyester fiber were 0.20% and 0.25%. The optimum content of hemp fiber was within the range of 0.20–0.25%. It could be seen from the water absorption and unconfined compressive strength tests that the polyester fiber was the most effective, followed by glass fiber, while hemp fiber proved to be the least effective. We therefore suggest that polyester fiber, which is a typical synthetic fiber, should be used to strengthen calcareous sand for optimal results. During the preparation of samples, the fibers easily formed into clumps, resulting in decreased uniformity of distribution of the fibers in the soil sample. Achieving a more even fiber distribution in the sand column was thus shown to greatly improve calcium carbonate distribution and soil sample strength improvement. These findings merit further investigation, analysis, and discussion in future research.

## Figures and Tables

**Table 1:** Physical and mechanical parameters of calcareous sand.

Property	Value
Specific gravity G_s_	2.76
Maximum dry density $\rho_{\max}$ (g/cm^3^)	1.524
Minimum dry density $\rho_{\min}$ (g/cm^3^)	1.230
d_10_ (mm)	0.26
d_30_ (mm)	0.42
d_60_ (mm)	0.58
Uniformity coefficient C_u_	2.231
Coefficient of curvature C_c_	1.170

**Table 2:** Physical and chemical properties of the fibers.

Fiber Types	Color	Length (mm)	Tensile Strength (MPa)	Density (g/cm^3^)	Modulus of Elasticity (GPa)	Fusion Point (°C)	Elongation (%)
Glass fiber	White	6	346	0.91	4.286	169	36.4
Polyester fiber	White	6	550	1.36	13.500	259	13.8
Hemp fiber	Yellow	6	255	1.12	5.369	189	26.6

**Table 3:** Test results of the biocemented sands with different fiber types and contents.

Group	Test No.	Fiber Content (%)	D_r_^a^ (%)	Δm ^b^ (g)	$\rho_{d}$^c^ (g/cm^3^)	*ω*^d^ (%)	UCS ^e^ (MPa)	Modulus of Elasticity (MPa)
Biocemented sand (BS)	BS	0	48	24.86	1.676	21.81	1.343	59.848
Glass fiber biocemented sand (GF-BS)	GF-BS-1	0.10	49	25.14	1.689	20.15	1.329	64.577
	GF-BS-2	0.15	50	26.73	1.706	19.99	1.448	50.121
	GF-BS-3	0.20	49	26.04	1.737	19.28	1.668	58.940
	GF-BS-4	0.25	48	24.58	1.684	22.11	1.593	61.553
	GF-BS-5	0.30	49	24.77	1.676	22.68	1.464	63.076
	GF-BS-6	0.35	49	24.02	1.654	22.62	1.253	56.929
	GF-BS-7	0.40	50	24.12	1.612	23.73	1.223	62.176
Polyester fiber biocemented sand (PF-BS)	PF-BS-1	0.10	49	26.33	1.707	21.37	1.502	66.080
	PF-BS-2	0.15	48	27.14	1.711	21.10	1.547	64.566
	PF-BS-3	0.20	50	27.31	1.724	18.67	1.729	64.732
	PF-BS-4	0.25	49	28.48	1.839	17.19	2.159	60.544
	PF-BS-5	0.30	50	25.42	1.742	20.58	1.645	66.626
	PF-BS-6	0.35	48	25.04	1.701	21.42	1.329	64.421
	PF-BS-7	0.40	49	25.26	1.707	21.23	1.184	63.147
Hemp fiber biocemented sand (HF-BS)	HF-BS-1	0.10	49	23.32	1.641	22.09	0.944	49.710
	HF-BS-2	0.15	50	23.97	1.649	22.27	0.980	54.657
	HF-BS-3	0.20	48	23.87	1.705	20.22	1.271	56.289
	HF-BS-4	0.25	49	25.84	1.657	22.01	1.429	60.397
	HF-BS-5	0.30	50	24.05	1.673	21.95	1.152	60.663
	HF-BS-6	0.35	50	24.36	1.640	22.51	0.975	63.976
	HF-BS-7	0.40	49	23.60	1.580	23.51	0.935	56.804

Note: a. Relative density ($D_{r}=\frac{(\rho_{d}{}^{\prime }-\rho_{\mathrm{dmin}})\rho_{\mathrm{dmax}}}{(\rho_{\mathrm{dmax}}-\rho_{\mathrm{dmin}})\rho_{d}{}^{\prime }}$ (where $\rho_{d}{}^{\prime }$ is the dry density before grouting, $\rho_{\mathrm{dmax}}$ is the maximum dry density before grouting, $\rho_{\mathrm{dmin}}$ is the minimum dry density before grouting); b. Calcium carbonate precipitation of the specimens ($\Delta m=m_{a}-m_{b}$ where $m_{a}$ is the dry mass after grouting, $m_{b}$ is the dry mass before grouting); c. Dry density after MICP treatment ($\rho_{d}=\frac{m_{a}}{V}$ where V is the volume of the cylinder sample); d. Water absorption of the specimens after grouting, as shown in Section 2.3.1. e. Unconfined Compressive Strength.

## Data Availability

The data presented in this study are available in J.Z., H.T., Y.S., J.Y., Q.P., J.L. Effects of Different Types of Fibers on the Physical and Mechanical Properties of MICP-Treated Calcareous Sand.
